# Stimulating the non-ARG harbored microbes with a specific carbon source can suppress the rebound of soil ARB after calcium peroxide application

**DOI:** 10.1016/j.fmre.2025.02.008

**Published:** 2025-02-26

**Authors:** Guoqing Niu, Shuiying Zhu, Xiaohang Yuan, Wenyi Yang, Zhexu Ding, Penghao Xie, Tao Wen, Qirong Shen, Jun Yuan

**Affiliations:** Jiangsu Provincial Key Lab for Organic Solid Waste Utilization, Jiangsu Collaborative Innovation Center for Solid Organic Wastes, Educational Ministry Engineering Center of Resource-saving fertilizers, Nanjing Agricultural University, Nanjing 210095, China

**Keywords:** Antibiotic-resistant bacteria, Soil physical and chemical properties, Calcium peroxide, D-Mannitol, Soil microbial community

## Abstract

The spread of antibiotic resistance genes poses a significant global environmental risk. Soil antibiotic-resistant bacteria (ARB) are a critical reservoir for antibiotic resistance genes in the environment, making them a focal point of concern. Traditional abatement techniques are not suitable for field applications at present. In this study, we investigated the removal of soil ARB using a combination of calcium peroxide and D-mannitol. Treatment with 2% CaO_2_ alone effectively reduced the populations of sulfonamide-resistant bacteria, beta-lactam-resistant bacteria, macrolide-resistant bacteria, and tetracycline-resistant bacteria by over 90% under a soil water holding capacity of 90%. However, this reduction decreased significantly after 40 days due to CaO_2_ depletion. To sustain the removal effect, we screened carbon sources and found that adding 1 mM D-mannitol 10 days after applying CaO_2_ could maintain removal rates of 78.41%, 91.44%, 86.81%, and 90.39% after 40 days. Sequencing results revealed that while CaO_2_ alone initially reduced ARB, it led to a rebound in genera such as *Ochrobactrum* and *Lysinibacillus*. In contrast, D-mannitol addition prevented this rebound and enriched non-ARB genera like *Nitrososphaera*, enhancing the overall removal effect. This study demonstrates that combining CaO_2_ with D-mannitol is a promising strategy for efficient and sustained removal of soil ARB.

## Graphical abstract

**Figure:**
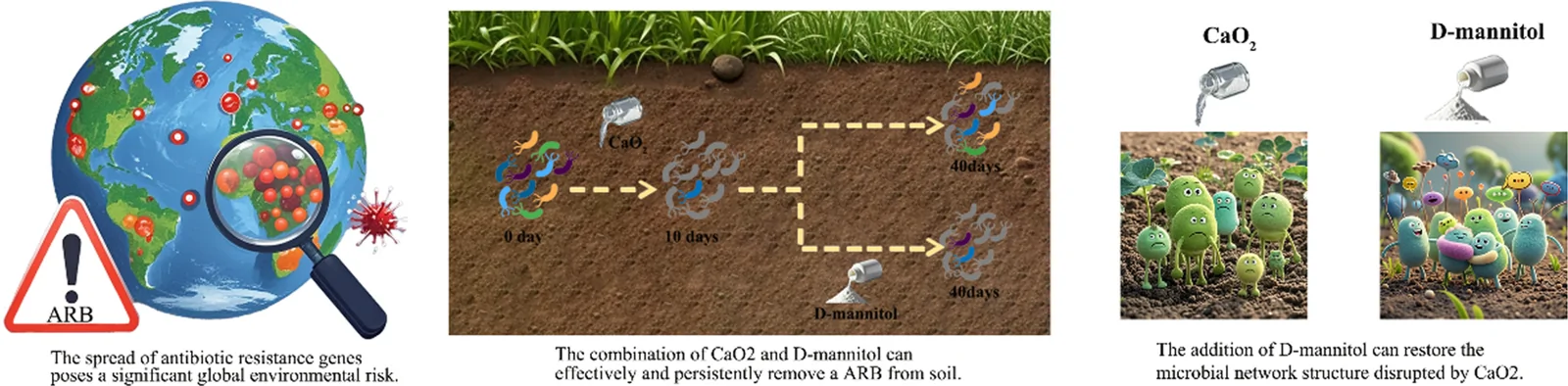

## 1 Introduction

In recent decades, the use of antibiotics in human medicine, veterinary practices, and livestock farming has markedly increased. However, the limited absorption of antibiotics by organisms means that more than half of the administered antibiotics are excreted through feces and subsequently enter the environment, particularly soil and water systems [1,2]. Studies have shown that excreted antibiotics can remain biologically active in soil for extended periods, posing ecological risks [3]. The primary concern with active antibiotics in the soil is the prolonged selective pressure on soil microorganisms, which can promote the evolution and enhancement of resistance mechanisms [4,5]. Microorganisms exposed to antibiotics may develop resistance through genetic mutations or the horizontal transfer of antibiotic resistance genes between plasmids [6,7], leading to the selective propagation of antibiotic-resistant bacteria (ARB) [8,9]. ARB present in soil can also infiltrate plants via the soil–plant pathway, potentially introducing these resistant bacteria into the food chain and posing health risks to higher-level consumers [[10], [11], [12]].

Currently, ARB is removed from soil predominantly through chemical oxidation technologies such as Fenton oxidation, chlorination disinfection, and ozone oxidation [13,14]. However, although these methods can significantly reduce ARB populations, the techniques require large quantities of hydrogen peroxide (H₂O₂), and the efficiency of H₂O₂ utilization in soil remediation is relatively low [15]. Therefore, developing cost-effective technologies to mitigate the spread of ARB in soil is essential. Calcium peroxide (CaO₂), commonly referred to as “solid” H₂O₂, is a stable oxidizing agent widely used for degrading pollutants owing to its strong oxidative properties [16]. For instance, Bao et al. demonstrated that adding CaO₂ to sewage sludge compost could simultaneously immobilize heavy metals and reduce antibiotic resistance genes [17]. CaO₂ particles gradually release H₂O₂ under moist conditions, generating highly oxidative hydroxyl radicals [18]. These radicals can disrupt enzymes, proteins, and DNA within microorganisms, and may damage microbial surface protection systems, causing an imbalance of intracellular and extracellular substances that leads to cell death [19]. For example, hydroxyl radicals produced by ultraviolet-activated persulfates were found to effectively remove ARB from wastewater, suggesting that CaO₂ could have potential in ARB remediation [20]. However, research indicates that even under intense anthropogenic disturbances, microbial communities can recover to their original states shortly after the removal of stress factors [21]. Consequently, when CaO₂ stress is alleviated, ARB in soil may regain activity and continue proliferating, potentially increasing their tolerance to CaO₂ over time. Therefore, establishing strategies to prevent the resurgence of ARB in soil is crucial.

From an ecological perspective, nutrient availability induces competition among soil microorganisms. When limiting nutrients are introduced, certain microbial groups may lose their competitive advantage and gradually undergo apoptosis due to nutrient restriction [22]. Hence, exogenous addition of specific limiting nutrients can influence microbial competition and reshape the soil microbial community structure [23]. Furthermore, by using species-specific resource preferences and providing carbon sources that beneficial microbes can metabolize but pathogens cannot, it is possible to selectively enrich beneficial microorganisms and suppress the growth of pathogenic microorganisms [24,25]. Inducing competition for ecological niches through the targeted addition of specific resources can result in a more diverse microbial community. This strategy has been widely applied in agricultural practices to regulate soil microbial composition and manage soil-borne diseases. For instance, malic acid has been shown to recruit *Bacillus subtilis* in *Arabidopsis thaliana* [26], fatty acids and amino acids promote the enrichment of *Pseudomonas* in soil [27], and L-sorbose or D-xylose facilitates the enrichment of *Klebsiella* with nitrite-accumulation functions [28]. These examples demonstrate that the addition of carbon sources can selectively enrich or deplete microorganisms based on their ability to utilize those carbon sources, thus influencing the composition of the microbial community [29,30]. Therefore, directly adding carbon sources to soil could be an approach for improving soil health or creating an environment that is unfavorable for the growth of ARB, thereby reducing the abundance of soil ARB.

In this study, we investigated the potential positive effects of removing four types of soil ARB—sulfonamide-resistant bacteria (SRB), macrolide-resistant bacteria (MRB), tetracycline-resistant bacteria (TRB), and beta-lactam-resistant bacteria (BRB)—through the combined application of CaO₂ and carbon sources. Research on the effectiveness of CaO₂ in eliminating soil ARB and the regulatory effects of supplemental carbon sources on ARB removal is limited. Therefore, we developed a novel strategy using soil culture experiments to evaluate the removal efficiency of the four types of soil ARB by CaO₂ and the potential benefits of subsequent carbon source supplementation. The main objectives of this study were: (1) to investigate the factors influencing the efficiency of CaO₂ in removing soil ARB; (2) to determine which carbon source could sustain ARB removal; and (3) to elucidate the microbial mechanisms underlying ARB removal through the combined application of CaO₂ and carbon sources.

## 2 Materials and methods

### 2.1 Applying and optimizing CaO_2_ for soil ARB removal

Top soil (0–20 cm) samples were collected around pig farms with several years of breeding history in Hai'an, China (120°45′9″ E, 32°54′51″ N). The physical and chemical properties of the soil are shown in Table S1. The soil was naturally air-dried and sieved (2-mm mesh sieve) before use.

The air-dried soil was used to establish a soil cultivation experiment, with three replicates for each experiment. Twenty grams of air-dried soil was evenly spread in a disposable culture dish, then 8 mL of sterile water was added and left for 24 h before tilting the culture dish to ensure no water flowed out; the amount of water added at this time was regarded as 100% water holding capacity (WHC) of the soil [31]. Different soil WHCs (70%, 80%, 90%, 100%, 110%) were set by adjusting the proportions of soil and water. Specifically, the amount of soil was constant at 20 g and 8.8 mL of sterile water was added for 110% WHC, 8 mL of sterile water was added for 100%, 7.2 mL of sterile water for 90%, 6.4 mL of sterile water for 80%, and 5.6 mL of sterile water for 70%. Simultaneously, different mass fractions (1%, 2%, 3%) of CaO_2_ were set by adding 0.2 g, 0.4 g, and 0.6 g of CaO_2_ to 20 g of soil, respectively, an untreated control with no added CaO_2_ was included. Uniform mixing of CaO_2_ with the soil was ensured using a sterile spoon. Throughout the experiment, strict control was maintained over the soil moisture, aiming to keep the soil WHC within a stable range. The disposable culture dishes were placed in a natural environment at 25 ± 2 °C for cultivation, and samples of the soil were taken at days 1 (2 h after application of CaO_2_), 10, 20, and 30 for ARB quantification with the plate counting method. After determining the optimal gradient of soil WHC, all subsequent experiments were conducted under this standard condition. In addition, to ensure the smooth release of H_2_O_2_ from CaO_2_, all experiments involving calcium peroxide were conducted under dark conditions.

The effect of different concentrations of CaO_2_ on the reduction of four types of soil ARB was investigated by weighing 20 g of air-dried soil into a disposable culture dish with 7.2 mL of sterile water, and then adding different mass fractions of CaO_2_ (1%, 2%, 3%). The specific experimental steps were as outlined above, with samples taken on days 1 (2 h after application of CaO_2_), 5, 10, 15, 20, 25, and 30 for ARB quantification via the plate counting method; a portion of each sample was also saved at −20 °C for further analysis.

The impact of different organic matter contents on the effectiveness of CaO_2_ in removing ARB was also examined. Three portions of 200 g of air-dried soil were individually weighed and placed in culture bottles. Subsequently, 0, 5, and 10 g of sterile organic fertilizer (organic matter content ≥ 45%, total nutrient (N + P_2_O_5_ + K_2_O) content ≥ 5%) were added to each portion, respectively, followed by 50 mL of sterile water for thorough mixing. The samples (labeled as soil1, soil2, and soil3, respectively) were then placed under natural conditions for 40 days to facilitate gradual integration between the organic fertilizer and the original soil particles [32]. The treated samples were air-dried and sieved, then 20 g of this air-dried soil was weighed into disposable culture dishes with 7.2 mL of sterile water, and then different mass fractions of CaO_2_ (1%, 2%, 3%) were added. The specific experimental steps are as above, with samples taken on day 1 (2 h after application of CaO_2_) for plate counting. Soil organic carbon (SOC) was measured using a carbon and nitrogen analyzer (Flash Smart, Thermo Fisher Scientific, USA). The organic matter content of Soil 1 was 38 g/kg, Soil 2 was 44 g/kg, and Soil 3 was 54 g/kg.

### 2.2 Plate counts of total culturable ARB

For bacterial plate counts, 1 g of soil was placed in a 50-mL sterile centrifuge tube containing 9 mL of sterile water. The tube was shaken for 20 min (200 rpm) on a shaker and allowed to settle for 5 min. Subsequently, 1 mL of the supernatant was transferred to a sterile clean test tube containing 9 mL of sterile water and serially diluted to 10^−5^. Next, 100 µL of the diluted samples were spread on beef paste peptone plates containing different antibiotics (sulfadiazine, ampicillin sodium, tetracycline, and tylosin). The concentrations of sulfadiazine, ampicillin sodium, tetracycline, and tylosin in the above media were 50, 50, 20, and 20 mg/L, respectively. The inoculated plates were incubated at 37 °C for 48 h, and then the total number of ARB colonies on the plates was counted [33]. *Escherichia coli* (DH5α), which is not resistant to any of the four included antibiotics, was spread on each antibiotic-containing plate as a negative control.

### 2.3 ARB isolation from soil samples and selection of the restrictive carbon source

To test the effects of CaO_2_ on the removal of ARB, we screened four types of ARB from soil samples treated with CaO_2_ (0%, 2%, and 3%) at three time points (days 1, 15, and 30), and 1200 colonies (100 colonies from each corresponding plate) were picked up and purified twice according to our previous method [34]. The isolated strains were mixed with 50% glycerol in a 1:1 ratio and stored long-term at −80 °C. For the classification and identification of each isolated strain, DNA was first extracted using a DNA extraction kit (B518255, Sengon Biotech, Shanghai), followed by concentration measurement using a NanoDrop 2000 spectrophotometer (Thermo Scientific, Waltham, MA, USA). PCR amplification was conducted using a two-step amplification method [34]. In the first step, full-length amplification was carried out with the primers 27F (5′-AGAGTTTGATCCTGGCTCAG-3′) and 1492R (5′-GGTTACCTTGTTACGACTT-3′). In the second step, the full-length PCR products served as templates for the amplification of the V4 region. The PCR program comprised pre-denaturation at 95 °C for 5 min, followed by 30 cycles of denaturation at 94 °C for 30 s, annealing at 55 °C for 25 s, and extension at 72 °C for 45 s, with a final extension at 72 °C for 5 min. The obtained PCR products were sequenced using the Illumina MiSeq platform (Magigene Biotech, Guangdong) for the V4 region of the 16S rRNA gene libraries. The sequencing results were analyzed according to Yuan et al [35].

Based on the sequencing results, eight representative ARB strains were selected. The Biolog GEN III microplate (Biolog, USA) was used to test the utilization of carbon sources by the eight strains, following the specific operation steps detailed in the product manual.

### 2.4 Combined application of CaO_2_ and D-mannitol for soil ARB removal and their effects on the soil microbiome

Carbon sources that were not utilized by any of the eight strains were identified through the Biolog GEN III microplate. Considering price and availability, D-mannitol was selected for combined application with CaO_2_ at three dosages. On the 10th day after applying CaO_2_, D-mannitol was added to the soil, resulting in concentrations of D-mannitol in the final soil of 0.01 µmol/g, 0.1 µmol/g, and 1 µmol/g. Samples without added D-mannitol but with the same volume of sterile water were included as a control treatment. All treated soils were incubated in a natural environment at 25 ± 2 °C. Soil samples were taken on days 15, 20, 25, 30, 35, and 40 for ARB counting. A portion of the soil was also stored at −20 °C until DNA extraction and further analysis.

Total genomic DNA was extracted from soil samples (0.5 g) using the PowerSoil DNA Isolation Kit (Qiagen, German) according to the manufacturer's instructions, and the quality and quantity of extracted DNA were measured using a NanoDrop 2000 spectrophotometer (Thermo Scientific, Waltham, MA, USA) and 2% agarose gel electrophoresis. The V4 region of the 16S rRNA gene library was sequenced using the Illumina MiSeq platform (Magigene Biotech, Guangdong). The bacterial primers 515 F (5′-GTGYCAGCMGCCGCGGTAA-3′) and 806 R (5′-GGACTACNVGGGTWTCTAAT-3′) were used for amplification [37]. The preparation of bacterial amplicon libraries and the high-throughput sequencing procedure followed the study by Yuan et al [35].

### 2.5 Analysis of the microbial community assembly processes

For community assembly processes, we used two methods to analyze the bacterial community phylogeny. First, the neutral model was applied to estimate the relative abundance of OTUs across the samples, and data fitting was performed to generate prediction intervals. Then, a null modeling approach was used to calculate the β-nearest taxon index (βNTI) and β-mean-nearest taxon distances (βMNTD) between sample pairs to assess phylogenetic patterns in the community, with Mantel tests used to evaluate the significance of βNTI values. To investigate the role of dispersal in community assembly, we examined OTU turnover using the Raup-Crick metric (RCbray), which assesses whether OTU turnover deviates from random ecological drift. By comparing observed Bray-Curtis dissimilarities with the null-model results, we calculated RCbray values and combined the results from βNTI and RCbray analyses to determine the relative proportions of deterministic versus stochastic processes governing community assembly [36].

### 2.6 Statistical analysis

Statistical analysis was performed using SPSS 26.0 statistical software (IBM, USA), with the method included one-way analysis of variance (ANOVA) (*P* < 0.05). The results were further visualized using Origin 9.0. Microbial analysis was conducted following previously published methods [38], and the results were visualized using the R package “ggplot2” [34]. Network analysis was performed using R package “ggClusterNet” [39].

## 3 Results

### 3.1 Effects of CaO_2_ on removing soil ARB

Considering that the antibacterial capacity of CaO_2_ is primarily from generating hydrogen peroxide (H_2_O_2_) when mixed with water, the WHC of tested soil on removal efficiency of ARB was evaluated. Higher soil WHC led to better removal effects of ARB by CaO_2_. However, when the soil WHC was greater than 90%, the addition of 1%, 2%, and 3% CaO_2_ had no significant impact on the removal efficiency of soil tetracycline-resistant bacteria (TRB), with the removal rate reaching 79.04%, 78.42%, and 83.39% after 40 days (Fig. 1A), respectively. Therefore, 90% WHC was selected as a fixed parameter for subsequent studies.

**Fig. 1:**
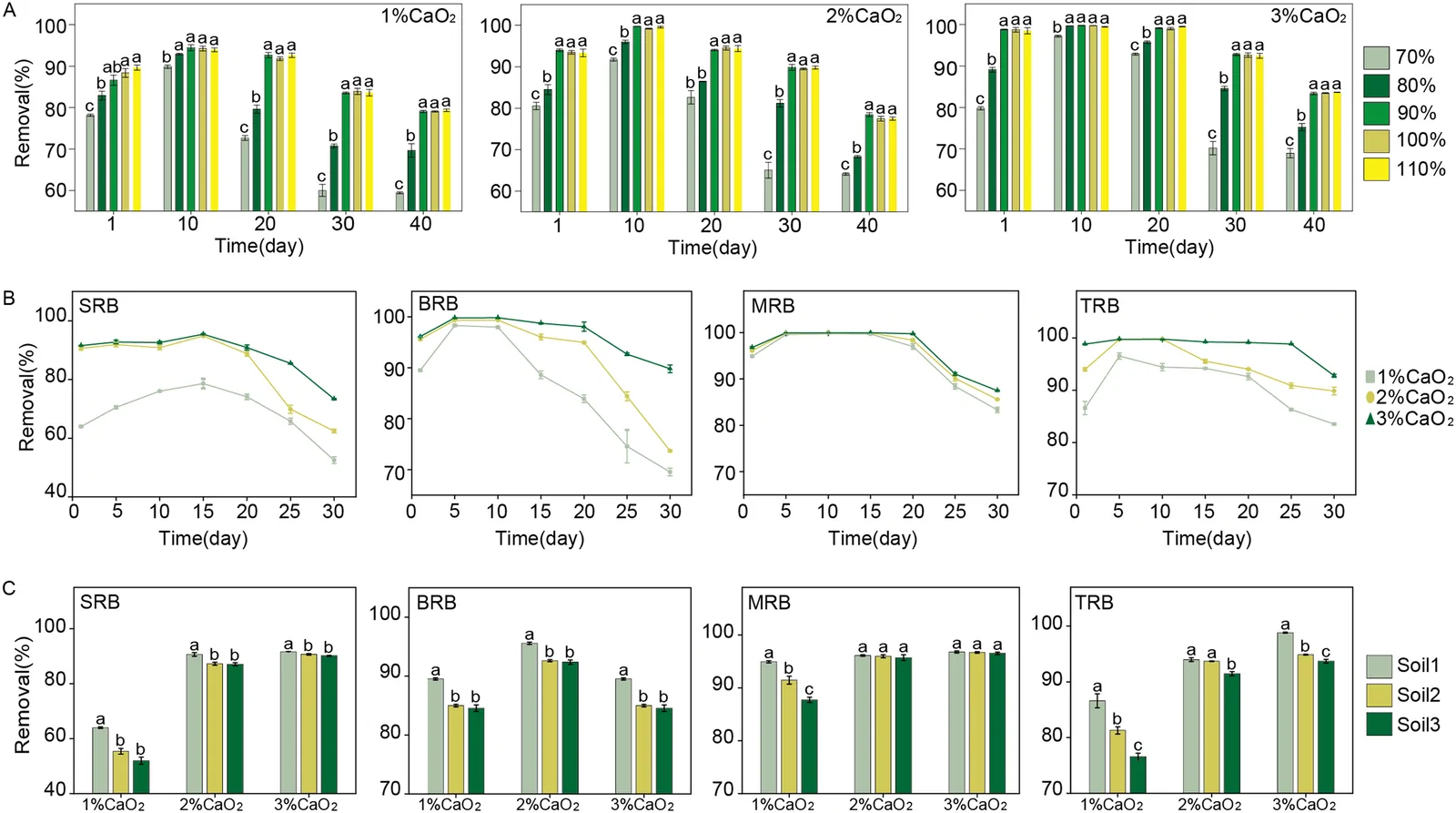
**Factors influencing the removal of soil antibiotic-resistant bacteria (ARB) by CaO₂.** CaO₂ was applied at three concentrations (1%, 2%, and 3%). WHC: Water Holding Capacity. (A) The three subplots represent the removal efficiencies of tetracycline-resistant bacteria (TRB) under 1%, 2%, and 3% CaO₂ concentrations, respectively, at different water holding capacities (WHC). (B) Effect of different amounts of CaO₂ on the removal of four types of soil ARB. (C) Effect of organic matter on the removal of four types of soil ARB by CaO₂: soil1, soil2, and soil3 represent soil samples with three different levels of soil organic matter. The four types of soil ARB are SRB (sulfonamide-resistant bacteria), TRB (tetracycline-resistant bacteria), MRB (macrolide-resistant bacteria), and BRB (beta-lactam-resistant bacteria).

The dosage of CaO_2_ was a crucial factor influencing the removal efficiencies of soil ARB. Overall, the removal efficiency of soil ARB was positively related to the CaO_2_ dosage. Over a period of 1 to 30 days, the removal efficiencies of four soil ARB exhibited an initial increase followed by a decline, peaking around days 10–15 (Fig. 1B). The removal efficiencies of the four soil ARB were not less than 60% with only 2% and 3% CaO_2_ addition on day 30 of the experiment. Therefore, there was a need to find a way to inhibit the rebound of soil ARB and maintain or promote the removal effect of CaO_2_, such as by adding carbon sources to regulate the bacterial community structure.

Soil organic matter (SOM) content can consume oxidants when oxidizing agents are used for soil remediation. Consequently, assessing the effect of SOM on the removal efficiency of ARB by CaO_2_ was necessary in this study. High SOM content decreased the removal efficiency of SRB, BRB, and TRB when CaO_2_ was at 1% to 3%. For MRB, the removal efficiency significantly decreased after 1% CaO_2_ treatment, but when CaO_2_ addition reached 2% and 3%, the SOM did not affect the removal efficiency of MRB by CaO_2_ (Fig. 1C). In addition, in the three soils with different SOM (soil1, soil2, and soil3), the addition of CaO_2_ significantly reduced SOM content compared with the treatment without CaO_2_, but there was no significant difference in SOM content among various CaO_2_ treatments (1%, 2%, 3%) (Fig. S1).

### 3.2 Identification of the soil ARB with high capacity of resilience after CaO_2_ treatment

To find soil ARB that were easily reactivated and had high abundance after depletion of CaO_2_, we isolated soil ARB in CaO_2_ treatments (0%, 2%, 3%) on days 1, 15, and 30. A total of 1200 colonies of four types of ARB were randomly selected and compared by 16S rRNA gene sequencing, yielding 177 strains of ARB from the screening of all soil samples. For soil SRB, the relative abundances of *Lysinibacillus, Pseudomonas*, and *Xanthomonadaceae* showed a decreasing trend on day 15 after 2% and 3% CaO_2_ treatments, but significantly increased on day 30 (Fig. 2A; Table S2). Similarly, for soil BRB, the relative abundances of *Bacillus, Brevundimonas*, and *Pseudoxanthomonas* decreased on day 15 after adding 2% and 3% CaO_2_, but increased on day 30 (Fig. 2B; Table S2). For soil MRB, the relative abundances of *Bacillus, Xanthomonadaceae, Ochrobactrum*, and *Lysinibacillus* significantly decreased on day 15 under 2% and 3% CaO_2_ treatments, but increased again on day 30 (Fig. 2C; Table S2). Likewise, for soil TRB, the relative abundances of *Bacillus, Ochrobactrum, Xanthomonadaceae*, and *Lysinibacillus* decreased on day 15 after the addition of 2% and 3% CaO_2_, but increased on day 30 (Fig. 2D; Table S2). In summary, regardless of whether treated with 2% or 3% CaO_2_, a clear rebound trend was observed in the relative abundance of some soil ARB on day 30 compared to day 15, including *Lysinibacillus, Ochrobactrum, Pseudomonas, Brevundimonas, Bacillus*, and *Xanthomonadaceae* (Figs. 2 and S2). Therefore, in subsequent experiments, we attempted to inhibit these easily rebounding soil ARB by adding restrictive carbon sources, in order to maintain or prolong the removal effect of CaO_2_ on ARB.

**Fig. 2:**
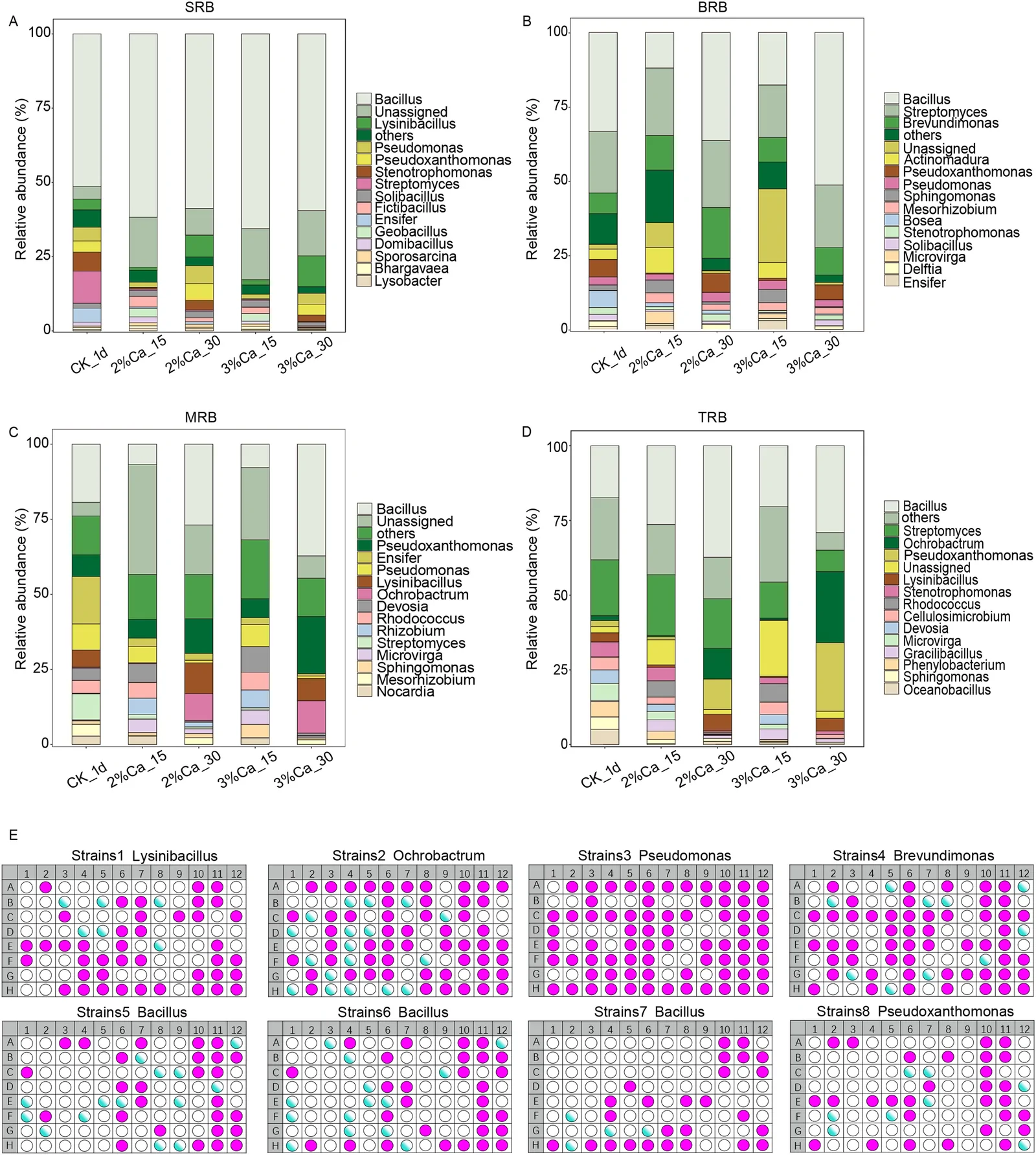
**Removal of antibiotic-resistant bacteria (ARB) by CaO**_**2**_**addition and selection of restrictive carbon sources.** Changes in the relative abundance of (A) SRB, (B) BRB, (C) MRB, and (D) TRB during 2% CaO_2_ treatment and 3% CaO_2_ treatment. (E) Carbon source utilization of eight ARB on the Biolog GEN III microplate. Well A-1 was used as a negative control. All wells that visually appeared similar to A-1 were designated “negative” (−); all wells that visibly appeared purple (darker than A-1) were positioned as “positive” (+). SRB: sulfonamide-resistant bacteria; TRB: tetracycline-resistant bacteria; MRB: macrolide-resistant bacteria; BRB: Beta-lactams-resistant bacteria.

### 3.3 Selection of restrictive (auxiliary) carbon sources

Limiting carbon sources were screened by selecting eight soil ARB that were easily restored and highly abundant at day 30 compared with day 15 as representative strain—including *Lysinibacillus, Ochrobactrum, Pseudomonas, Brevundimonas, Bacillus*(3 strains), and *Pseudoxanthomonas*—and observing their carbon source utilization through the Biolog GEN III microplate, where the carbon sources were laid out in the layout of the reference Fig. S3. The color development results showed that p-hydroxy-phenylacetic acid, β-methyl-D-glucoside, D-mannitol, and D-aspartic acid could not be utilized by the eight soil ARB (Fig. 2E). Considering the toxicity, availability, and cost of these restrictive carbon sources, D-mannitol was selected for further investigation (Table S3).

### 3.4 Effect of D-mannitol on soil ARB removal by CaO_2_

The addition of D-mannitol at day 10 after CaO_2_ treatment significantly enhanced the removal efficiency of four soil ARB (Fig. 3A-B). Specifically, under the 2% CaO_2_ treatment, the removal rates of soil BRB and TBR were 66.80% and 78.76% after 40 days, respectively. Compared with the treatment without D-mannitol, the addition of 0.1, 1, and 10 mM D-mannitol significantly increased the removal rates of soil BRB by 16.18%, 24.64%, and 25.99%, respectively, and those of soil TRB by 7.38%, 11.64%, and 11.29%, respectively. For both of these soil ARB types, there was no significant difference between 1 mM and 10 mM D-mannitol application (Fig. 3C). A similar pattern was observed for soil BRB and TRB under the 3% CaO_2_ treatment (Fig. 3D). Therefore, 1 mM was selected as the optimal concentration of D-mannitol. In addition, when the concentration of D-mannitol was 1 mM, the addition of 2% CaO_2_ and 3% CaO_2_ had no significant effects on the removal rates of soil SRB, MRB, and TRB at day 40 (*P* > 0.01) (Fig. S3). Therefore, we selected the combination of 2% CaO_2_ and 1 mM D-mannitol as the optimal approach for removing soil ARB.

**Fig. 3:**
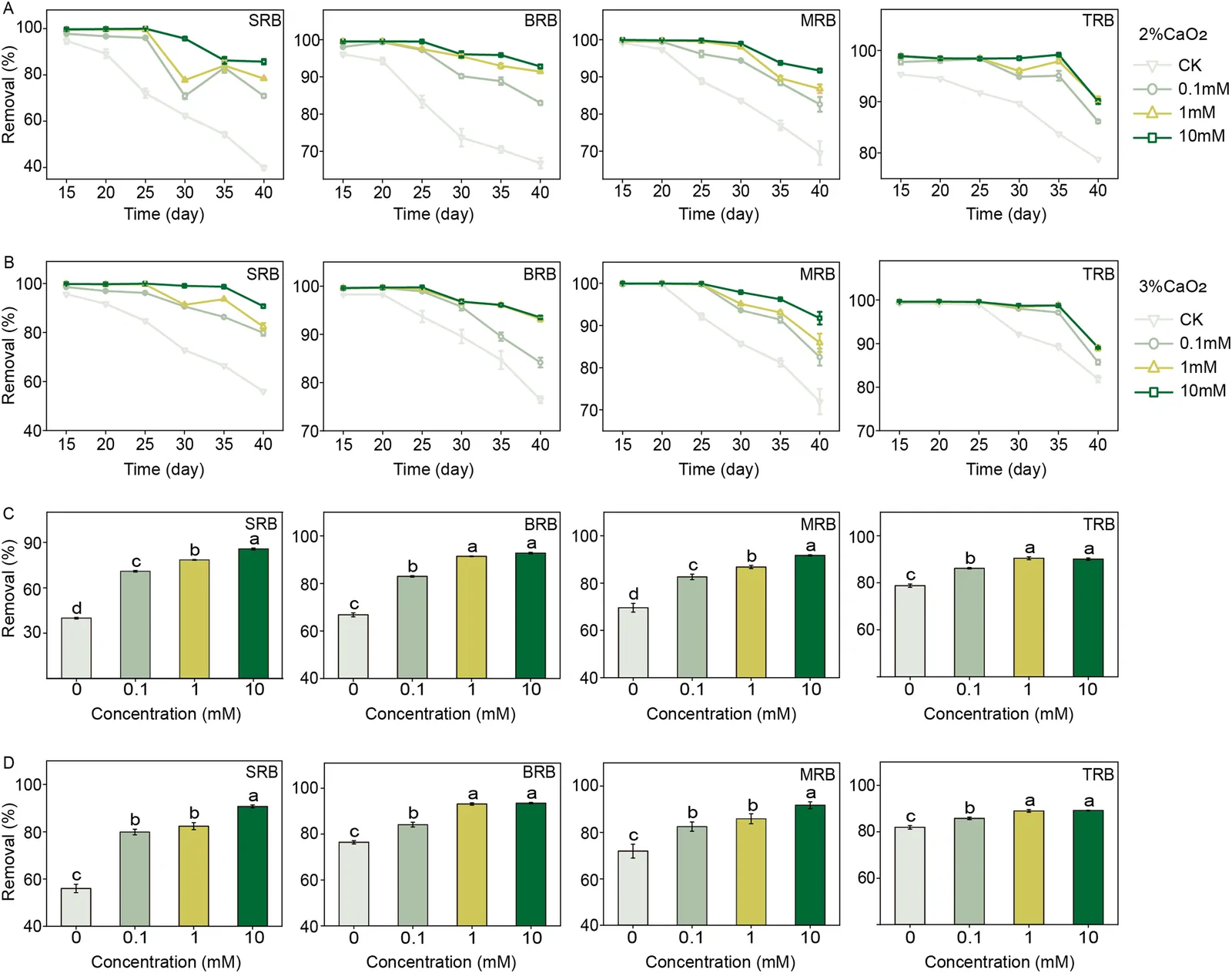
**Removal of four soil antibiotic-resistant bacteria (ARB) by CaO**_**2**_**treatment for 10 days followed by addition of different concentrations of D-mannitol.** (A) 2% CaO_2_ treatment. (B) 3% CaO_2_ treatment. (C) Removal rates of soil ARB by 2% CaO_2_ treatment with different concentrations of D-mannitol on day 40 of the experiment. (D) Removal rates of ARB by 3% CaO_2_ treatment with different concentrations of D-mannitol on the day 40 of the experiment. The different concentrations of D-mannitol used in the experiment were 0, 0.1, 1, and 10 mM. SRB: sulfonamide- resistant bacteria; TRB: tetracycline-resistant bacteria; MRB: macrolide-resistant bacteria; BRB: Beta-lactam-resistant bacteria.

### 3.5 Changes in bacterial community composition

Compared with the control (CK), the alpha diversity of soil bacterial communities was significantly lower among all treatments. In addition, compared with the 2% CaO_2_ treatment for 10 days (Ca_10), a significant decrease in the Shannon index was observed in the CaO_2_ treatment for 20 days (Ca_20) and the CaO_2_ treatment for 30 days (Ca_30), but no significant change was found in the Shannon index in the treatment of 2% CaO_2_ combined with 1 mM D-mannitol for 20 days (Ca_M_20) and the treatment of 2% CaO_2_ combined with 1 mM D-mannitol for 30 days (Ca_M_30) (Fig. 4A). The PCoA with Bray–Curtis distance matrix revealed that CaO_2_ significantly affected the distribution of soil bacterial communities (ANOSIM.r = 0.805, *P* = 0.001) (Fig. 4B). The distance among Ca_10, Ca_M_20, and Ca_M_30 was relatively close, indicating that the addition of D-mannitol maintained the community structure after CaO_2_ treatment for 10 days. Analysis of the community composition showed that there were significant differences between treatments compared with the control soil (Fig. 4C). Specifically, compared with CK treatment, the relative abundance of *Ochrobactrum, Lysinibacillus, Aneurinibacillus, GP7, Ohtaekwangia*, and *Subdivision3_genera_incertae_sedis* were significantly decreased in the Ca_10 treatment, but the relative abundance of these genera was significantly increased in the Ca_30 treatment. This indicated that CaO_2_ can effectively change the composition of the soil bacterial community, but the inhibited soil ARB become active again after CaO_2_ depletion. Compared with Ca_10 treatment, we found that the species composition of the Ca_M_20 and Ca_M_30 treatments were similar to that of the Ca_10 treatment at the genus level, although the relative abundance of *Bacillus* and *Pseudomonas* increased slightly, the relative abundance of some species such as *Ochrobactrum, Lysinibacillus, Aneurinibacillus, Azoarcus*, and *Ohtaekwangia* showed no significant changes. This indicated that adding D-mannitol effectively alleviated the rebound of soil bacteria after CaO_2_ depletion. Furthermore, compared with Ca_10 treatment, adding D-mannitol significantly increased the relative abundance of *Nitrososphaera, GP10*, and *GP4* on day 30 of the experiment, and these three types of bacteria were not present in the random screening sequencing results (Fig. S2). This indicated that D-mannitol enriched non-soil ARB and enhanced the removal effect of CaO_2_.

**Fig. 4:**
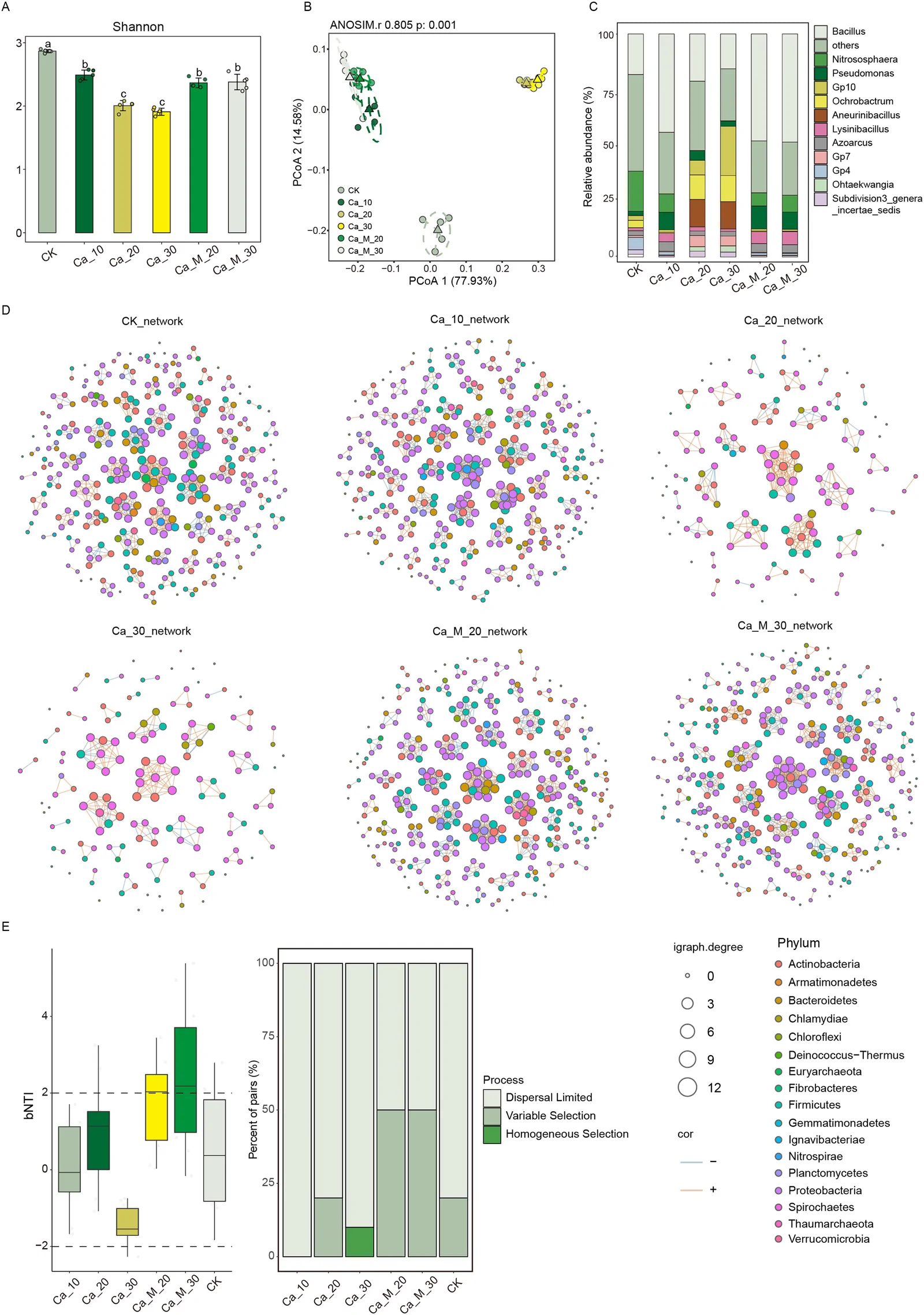
**Effects of 1 mM D-mannitol added after 10 days of 2% CaO**_**2**_**treatment on the soil bacterial community.** (A) Alpha diversity of the soil bacterial communities across the different treatments. Shannon calculated using the normalized ASV table. (B) Principal coordinate analysis (PCoA) with Bray–Curtis dissimilarity performed the CaO_2_ affected the distribution of soil bacterial communities. (C) Relative abundance (%) of the major genera in bacteria communities. (D) Network analysis of different treatments. (E) Contributions of deterministic and stochastic processes in bacterial community assembly within different treatments. CK represents the original soil; Ca_10 represents 2% CaO_2_ treatment for 10 days; Ca_20 represents 2% CaO_2_ treatment for 20 days; Ca_30 represents 2% CaO_2_ treatment for 30 days; Ca_M_20 represents the treatment of 2% CaO_2_ combined with 1 mM D-mannitol for 20 days; Ca_M_30 represents the treatment of 2% CaO_2_ combined with 1 mM D-mannitol for 30 days.

The microbial interactions in each treatment were further explored through the co-occurrence network (Fig. 4D). The results of network topology comparison showed that compared with the Ca_10 treatment, the proportion of negative edges was lower in the Ca_20 and Ca_30 treatments. However, the addition of D-mannitol (Ca_M_20 and Ca_M_30) did not significantly change the proportion of negative edges (Fig. 4D), indicating that D-mannitol could maintain the stability of the soil bacterial network after adding CaO_2_.

To investigate the role of D-mannitol in the treatment process, we analyzed the assembly of soil bacterial communities under different treatments. The β-nearest taxon index (βNTI) was employed to evaluate the community assembly process (Fig. 4E). Stochastic processes dominated the assembly in all soil bacterial communities without D-mannitol (|βNTI| < 2). In contrast, the βNTI value exceeded 2 in the D-mannitol treatment, indicating that deterministic processes influenced the bacterial community assembly. Furthermore, RC-Bray analysis revealed that bacterial communities in the initial soil and those treated with CaO_2_ alone were primarily affected by limited dispersal. However, in the combined treatment of CaO_2_ and D-mannitol, the bacterial community assembly was predominantly governed by variable selection (Fig. 4E).

## 4 Discussion

Soil is the foundation of human society, as it supports 95% of the food for human consumption [40]. However, polluted soil poses a risk to humans. Recently, the continuous application of antibiotics in livestock farming has led to local soil contamination with concentrated antibiotic(s), resulting in the enrichment of ARB [1]. Moreover, there is concern that the ARB in soil may easily transfer to plants and even migrate to humans through the food chain, posing a potential threat to public health [41].

Calcium peroxide (CaO_2_) is non-toxic and environmentally friendly, making it a suitable oxidant for various environmental applications such as wastewater treatment and sediment treatment [[42], [43], [44]]. In sewage sludge composting, CaO₂ treats heavy metals and removes antibiotic-resistance genes [17]. CaO₂ releases H_2_O_2_ in dark and moist conditions, generating highly oxidative hydroxyl radicals that effectively remove ARB from wastewater [[18], [19], [20]]. In our study, CaO₂ was considered an effective approach for addressing soil ARB contamination under certain conditions. Therefore, determining the optimal application conditions of CaO₂ is crucial for maximizing the efficiency of the treatment. Soil moisture can affect the interaction between CaO_2_ and soil microorganisms. Previous studies indicated that increasing soil WHC can enhance the interface between the oxidizer and the soil, thereby improving the effectiveness of the oxidizer [45]. Our findings also demonstrate that the removal rate of soil ARB by CaO_2_ gradually increases as the soil WHC increases. In other words, when the soil WHC was low, there may be uneven mixing of CaO_2_ and soil, but as the soil WHC increased, CaO_2_ was more likely to diffuse and migrate in the soil along with water molecules. However, once the soil WHC exceeded 90%, the reduction rate of soil ARB by CaO_2_ no longer increased. This phenomenon may be attributed to the complete contact between CaO_2_ and soil after reaching the optimal soil WHC. Moreover, excessive soil WHC may lead to the depletion of CaO₂—a finding that has been confirmed in similar studies using CaO₂ for soil pollutant remediation [46].

Simultaneously, SOM is an important indicator of soil fertility. Studies have shown that the formation and transformation of free radicals in soil-containing media were affected by SOM. Jiang et al. found that soil depth significantly affected the degradation rate of s-triazine herbicides by oxidants, noting substantial variations in SOM across different soil profiles and observing a decrease in degradation efficiency as SOM content increased [47]. Our results showed that the removal rate of soil ARB was significantly reduced with higher SOM, especially for the 1% CaO_2_ treatment. A possible explanation is that SOM—acting as a reducing or bursting agent—consumed most of the reactive substances, such as hydroxyl radicals, in soil with a high SOM content, thereby decreasing the removal rate of soil ARB by CaO_2_ [19,48,49]. The significant decrease in SOM after CaO_2_ treatment further supports the idea that SOM interacts with CaO_2_, affecting the rate of ARB removal. Our results also indicated that increasing the addition of CaO_2_ can improve the removal rate of the four selected soil ARB, excessive amounts did not lead to a significant improvement, which may be due to CaO_2_ relying on reactive oxygen species for sterilization, and a small number of microorganisms in soil were passively stored in soil aggregates or soil particles [50]. The interior of the soil aggregates potentially provided a unique heterogeneous microhabitat to physically protect some microorganisms, thus avoiding exposure of soil ARB to CaO_2_. Certini *et al*. showed that soil particles can protect bacteria and cope with unfavorable stresses [51]. Another possible explanation is that an excessive amount of CaO_2_ killed most of the microorganisms in the soil, leading to a decrease in microbial activity [43,49]. Meanwhile, the initial decrease followed by an increase in the number of soil ARB after CaO_2_ addition may be attributed to the strong recovery ability of soil microbial communities, even under intense disturbances caused by human activities [52]. In other words, CaO_2_ in the soil was consumed during the later stages of our experiment, and the ecosystems began to regain the activity of the remaining soil ARB after the oxidative stress was relieved.

To inhibit the reactivation of soil ARB and maintain or enhance the removal of CaO_2_, we start from the perspective of resource allocation in ecological niches [53]. Ecologically, nutrient resources create competition for soil microorganisms, and resource utilization and preference are key factors influencing ecological niches. With the addition of limiting resources, specific microbial taxa in the soil may lose their ecological niche and perish due to nutrient limitation [54,55]. Therefore, we attempted to inhibit the rebound and enrichment of soil ARB in this study by adding restrictive carbon sources, aiming to maintain the removal effect of CaO_2_ on soil ARB. Previous studies of carbon sources have focused on recruiting beneficial microbes to inhibit the growth of pathogenic microbes [56]. Yang et al. investigated how different types and abundances of carbon sources affect the competitive interactions between *Bacillus amyloliquefaciens T-5* and *Ralstonia solanacearum* [57]. In contrast, our study provided a different approach of using carbon sources to “starve” certain soil ARB. Through amplification sequencing technology, we identified representative soil ARB that were easily reactivated after CaO_2_ depletion. Then, based on the colorimetric results from the Biolog GEN III microplate and a comprehensive evaluation of the restrictive carbon sources’ toxicity, availability, and cost, only D-mannitol meets the screening criteria due to its easy availability, low toxicity, and cost-effectiveness. Therefore, we selected D-mannitol as the restrictive carbon source for the experiment. The results of soil incubation experiments showed that the addition of D-mannitol effectively maintained and improved the removal effect of CaO_2_ on four soil ARBs (Fig. 3). In addition, the soil bacterial community structure changed significantly with CaO_2_ application between day 1 and 30 of the experiment when compared with CK treatment. Although the relative abundance of some bacterial genera decreased significantly on day 10 and then increased again on day 30 of the experiment, such as *Ochrobactrum* and *Lysinibacillus*, the relative abundance of these bacterial genera did not exhibit a rebound trend in the combined treatment of CaO_2_ and D-mannitol on day 30 of the experiment (Fig. 4C). The addition of D-mannitol may have altered the distribution of resources in the soil, reducing the ecological niches available to ARB and thereby effectively suppressing their rebound, maintaining the removal efficiency of CaO₂. Experimental results show that adding D-mannitol prevented the rebound of ARB and significantly enriched non-ARB genera such as *Nitrososphaera, GP10*, and *GP4*. This phenomenon may be related to the selective effects of D-mannitol, which provided a competitive advantage for specific microorganisms, promoting their growth and enhancing their adaptability and competitiveness in the soil ecosystem (Figs. 2 and S2).

Our study also demonstrated that the combined treatment of CaO₂ and D-mannitol effectively reduced the relative abundance of most microorganisms and could even sustain and enhance this effect. In addition, when 2% CaO_2_ combined with 1 mM D-mannitol was applied for 30 days (Ca_M_30), the relative abundance of *Bacillus* and *Pseudomonas* remained high, which may be its strong ecological competitiveness [58,59]. Qiu et al. showed that erosion can reduce the diversity, network complexity, and versatility of soil microorganisms [60]. In this study, we observed that adding CaO_2_ destroyed the soil microbial network pattern, which may cause the soil ecosystem to collapse more easily. However, the combined application of D-mannitol and CaO₂ not only enhanced the removal efficiency of CaO₂ but also suppressed the rebound of ARB and enriched non-ARB genera, maintaining the stability of the soil microbial community and preventing the collapse of the ecological network (Fig. 4D). This mechanism suggests that D-mannitol is a key supplementary strategy for achieving long-term, efficient removal of soil ARB. Furthermore, with CaO_2_ addition, stochastic processes dominated in the assembly of all soil bacterial communities. However, deterministic processes dominated with application of D-mannitol. D-Mannitol released the selective pressure exerted by CaO_2_ on soil bacterial community assembly. This further demonstrated that the application of D-mannitol following CaO₂ promoted the formation of a soil community with a reduced abundance of ARB.

## 5 Conclusion

Our study provides a new soil remediation method—CaO_2_ combined with a restrictive carbon source (D-mannitol)—for removing soil ARB. This method predominantly inhibits and removes soil ARB by utilizing the strong oxidizing properties of CaO_2_ and altering the ecological niche of soil microorganisms through the addition of a carbon source. Treatment with 2% CaO_2_ for 10 days, followed by the addition of 1 mM D-mannitol significantly increased and prolonged the removal efficiency of SRB, MRB, BRB, and TRB in soil by CaO_2_. Moreover, the addition of D-mannitol maintained the soil bacterial composition after 10 days of CaO_2_ treatment, ensuring the high stability of the soil ecosystem. In conclusion, the method proposed in this study provides a feasible solution for addressing the excessive presence of ARB in soil.

## Data availability

All data are available in the main text or the supplementary materials.

## Declaration of competing interest

The authors declare that they have no conflicts of interest in this work.

## Appendix Supplementary materials

Image, application 1
