# Supplementary material for: Stimulating the non-ARG harbored microbes with a specific carbon source can suppress the rebound of soil ARB after calcium peroxide application
Source: Fundam Res. 2025 Feb 26;6(5):3095–103. doi: 10.1016/j.fmre.2025.02.008 (PMC13644412; doi:10.1016/j.fmre.2025.02.008)
Supplement: Supplementary file 1 [file mmc1.docx]

**Supplementary information for**

**Stimulating the non-ARG harbored microbes with specific carbon source can suppressive the rebound of soil ARB after calcium peroxide application**

Guoqing Niu^#^, Shuiying Zhu^#^, Xiaohang Yuan, Wenyi Yang, Zhexu Ding, Penghao Xie, Tao Wen, Qirong Shen, Jun Yuan *

**Table. S1: Physical and chemical properties of the original soil**

| pH | NO^3-^(mg/kg) | NH^4+^  (mg/kg) | Available P (mg/kg) | Available K (mg/kg) |
| --- | --- | --- | --- | --- |
| 7.86±0.02 | 10.5 | 30.1 | 204.0 | 707.0 |

**Table. S2: Changes in the relative abundance of ARBs in 4 types of soils under 2% and 3% calcium peroxide treatments.**

| SRB | | |
| --- | --- | --- |
| CK_1d | Bacillus | 51.32597 |
| CK_1d | Streptomyces | 10.77348 |
| CK_1d | Stenotrophomonas | 6.40884 |
| CK_1d | others | 5.856354 |
| CK_1d | Pseudomonas | 4.640884 |
| CK_1d | Ensifer | 4.640884 |
| CK_1d | Unassigned | 4.309392 |
| CK_1d | Pseudoxanthomonas | 3.756906 |
| CK_1d | Lysinibacillus | 3.59116 |
| CK_1d | Solibacillus | 1.657459 |
| CK_1d | Domibacillus | 1.270718 |
| CK_1d | Bhargavaea | 0.773481 |
| CK_1d | Sporosarcina | 0.441989 |
| CK_1d | Lysobacter | 0.441989 |
| CK_1d | Geobacillus | 0.055249 |
| CK_1d | Fictibacillus | 0.055249 |
| 2%Ca_15 | Bacillus | 61.72662 |
| 2%Ca_15 | Unassigned | 16.83453 |
| 2%Ca_15 | others | 4.028777 |
| 2%Ca_15 | Fictibacillus | 3.597122 |
| 2%Ca_15 | Geobacillus | 2.733813 |
| 2%Ca_15 | Domibacillus | 2.014388 |
| 2%Ca_15 | Solibacillus | 2.014388 |
| 2%Ca_15 | Pseudomonas | 1.726619 |
| 2%Ca_15 | Lysinibacillus | 1.007194 |
| 2%Ca_15 | Sporosarcina | 1.007194 |
| 2%Ca_15 | Bhargavaea | 0.863309 |
| 2%Ca_15 | Lysobacter | 0.863309 |
| 2%Ca_15 | Ensifer | 0.57554 |
| 2%Ca_15 | Stenotrophomonas | 0.431655 |
| 2%Ca_15 | Streptomyces | 0.431655 |
| 2%Ca_15 | Pseudoxanthomonas | 0.143885 |
| 2%Ca_30 | Bacillus | 58.76841 |
| 2%Ca_30 | Unassigned | 8.96921 |
| 2%Ca_30 | Lysinibacillus | 7.362784 |
| 2%Ca_30 | Pseudomonas | 6.024096 |
| 2%Ca_30 | Pseudoxanthomonas | 5.62249 |
| 2%Ca_30 | Stenotrophomonas | 3.212851 |
| 2%Ca_30 | others | 2.945114 |
| 2%Ca_30 | Solibacillus | 2.27577 |
| 2%Ca_30 | Fictibacillus | 1.338688 |
| 2%Ca_30 | Sporosarcina | 1.07095 |
| 2%Ca_30 | Ensifer | 0.803213 |
| 2%Ca_30 | Lysobacter | 0.803213 |
| 2%Ca_30 | Bhargavaea | 0.401606 |
| 2%Ca_30 | Streptomyces | 0.401606 |
| 2%Ca_30 | Domibacillus | 0 |
| 2%Ca_30 | Geobacillus | 0 |
| 3%Ca_15 | Bacillus | 65.58266 |
| 3%Ca_15 | Unassigned | 17.20867 |
| 3%Ca_15 | others | 3.116531 |
| 3%Ca_15 | Geobacillus | 2.574526 |
| 3%Ca_15 | Solibacillus | 2.303523 |
| 3%Ca_15 | Fictibacillus | 2.168022 |
| 3%Ca_15 | Lysinibacillus | 1.761518 |
| 3%Ca_15 | Pseudomonas | 1.490515 |
| 3%Ca_15 | Domibacillus | 0.948509 |
| 3%Ca_15 | Bhargavaea | 0.948509 |
| 3%Ca_15 | Sporosarcina | 0.813008 |
| 3%Ca_15 | Lysobacter | 0.542005 |
| 3%Ca_15 | Streptomyces | 0.271003 |
| 3%Ca_15 | Stenotrophomonas | 0.135501 |
| 3%Ca_15 | Pseudoxanthomonas | 0.135501 |
| 3%Ca_15 | Ensifer | 0 |
| 3%Ca_30 | Bacillus | 59.52133 |
| 3%Ca_30 | Unassigned | 15.19251 |
| 3%Ca_30 | Lysinibacillus | 10.40583 |
| 3%Ca_30 | Pseudomonas | 3.746098 |
| 3%Ca_30 | Pseudoxanthomonas | 3.64204 |
| 3%Ca_30 | Stenotrophomonas | 2.185224 |
| 3%Ca_30 | others | 2.185224 |
| 3%Ca_30 | Solibacillus | 1.352758 |
| 3%Ca_30 | Bhargavaea | 0.416233 |
| 3%Ca_30 | Fictibacillus | 0.416233 |
| 3%Ca_30 | Sporosarcina | 0.416233 |
| 3%Ca_30 | Ensifer | 0.312175 |
| 3%Ca_30 | Lysobacter | 0.104058 |
| 3%Ca_30 | Streptomyces | 0.104058 |
| 3%Ca_30 | Domibacillus | 0 |
| 3%Ca_30 | Geobacillus | 0 |

| BRB | | |
| --- | --- | --- |
| CK_1d | Bacillus | 33.30619 |
| CK_1d | Streptomyces | 20.72476 |
| CK_1d | others | 10.26059 |
| CK_1d | Brevundimonas | 7.003257 |
| CK_1d | Pseudoxanthomonas | 5.863192 |
| CK_1d | Bosea | 5.659609 |
| CK_1d | Actinomadura | 3.542345 |
| CK_1d | Pseudomonas | 2.728013 |
| CK_1d | Stenotrophomonas | 2.320847 |
| CK_1d | Solibacillus | 2.076547 |
| CK_1d | Sphingomonas | 1.832248 |
| CK_1d | Delftia | 1.710098 |
| CK_1d | Unassigned | 1.587948 |
| CK_1d | Ensifer | 1.058632 |
| CK_1d | Microvirga | 0.2443 |
| CK_1d | Mesorhizobium | 0.081433 |
| 2%Ca_15 | Streptomyces | 22.66667 |
| 2%Ca_15 | others | 17.66667 |
| 2%Ca_15 | Bacillus | 12 |
| 2%Ca_15 | Brevundimonas | 11.66667 |
| 2%Ca_15 | Actinomadura | 8.666667 |
| 2%Ca_15 | Unassigned | 8.333333 |
| 2%Ca_15 | Sphingomonas | 4.333333 |
| 2%Ca_15 | Microvirga | 4 |
| 2%Ca_15 | Mesorhizobium | 3.333333 |
| 2%Ca_15 | Pseudomonas | 2 |
| 2%Ca_15 | Bosea | 1.333333 |
| 2%Ca_15 | Ensifer | 1.333333 |
| 2%Ca_15 | Stenotrophomonas | 1 |
| 2%Ca_15 | Delftia | 0.666667 |
| 2%Ca_15 | Solibacillus | 0.666667 |
| 2%Ca_15 | Pseudoxanthomonas | 0.333333 |
| 2%Ca_30 | Bacillus | 36.33491 |
| 2%Ca_30 | Streptomyces | 22.59084 |
| 2%Ca_30 | Brevundimonas | 17.06161 |
| 2%Ca_30 | Pseudoxanthomonas | 6.477093 |
| 2%Ca_30 | others | 4.107425 |
| 2%Ca_30 | Pseudomonas | 3.159558 |
| 2%Ca_30 | Stenotrophomonas | 2.369668 |
| 2%Ca_30 | Mesorhizobium | 2.053712 |
| 2%Ca_30 | Delftia | 1.579779 |
| 2%Ca_30 | Bosea | 1.263823 |
| 2%Ca_30 | Solibacillus | 0.947867 |
| 2%Ca_30 | Actinomadura | 0.789889 |
| 2%Ca_30 | Sphingomonas | 0.789889 |
| 2%Ca_30 | Ensifer | 0.157978 |
| 2%Ca_30 | Microvirga | 0.157978 |
| 2%Ca_30 | Unassigned | 0.157978 |
| 3%Ca_15 | Unassigned | 24.81203 |
| 3%Ca_15 | Bacillus | 17.66917 |
| 3%Ca_15 | Streptomyces | 17.66917 |
| 3%Ca_15 | others | 9.022556 |
| 3%Ca_15 | Brevundimonas | 8.270677 |
| 3%Ca_15 | Actinomadura | 5.263158 |
| 3%Ca_15 | Sphingomonas | 4.511278 |
| 3%Ca_15 | Ensifer | 3.007519 |
| 3%Ca_15 | Pseudomonas | 3.007519 |
| 3%Ca_15 | Mesorhizobium | 2.631579 |
| 3%Ca_15 | Microvirga | 1.503759 |
| 3%Ca_15 | Delftia | 0.75188 |
| 3%Ca_15 | Pseudoxanthomonas | 0.75188 |
| 3%Ca_15 | Stenotrophomonas | 0.75188 |
| 3%Ca_15 | Solibacillus | 0.37594 |
| 3%Ca_15 | Bosea | 0 |
| 3%Ca_30 | Bacillus | 51.30971 |
| 3%Ca_30 | Streptomyces | 21.1094 |
| 3%Ca_30 | Brevundimonas | 9.244992 |
| 3%Ca_30 | Pseudoxanthomonas | 4.930663 |
| 3%Ca_30 | others | 2.465331 |
| 3%Ca_30 | Pseudomonas | 2.311248 |
| 3%Ca_30 | Mesorhizobium | 2.157165 |
| 3%Ca_30 | Solibacillus | 2.003082 |
| 3%Ca_30 | Stenotrophomonas | 1.694915 |
| 3%Ca_30 | Delftia | 1.078582 |
| 3%Ca_30 | Unassigned | 0.616333 |
| 3%Ca_30 | Actinomadura | 0.308166 |
| 3%Ca_30 | Bosea | 0.308166 |
| 3%Ca_30 | Sphingomonas | 0.308166 |
| 3%Ca_30 | Ensifer | 0.154083 |
| 3%Ca_30 | Microvirga | 0 |

| TRB | | |
| --- | --- | --- |
| CK_1d | others | 20.81247 |
| CK_1d | Streptomyces | 18.69783 |
| CK_1d | Bacillus | 17.36227 |
| CK_1d | Microvirga | 5.787423 |
| CK_1d | Phenylobacterium | 5.175292 |
| CK_1d | Oceanobacillus | 5.119644 |
| CK_1d | Stenotrophomonas | 5.008347 |
| CK_1d | Devosia | 4.451864 |
| CK_1d | Cellulosimicrobium | 4.229271 |
| CK_1d | Sphingomonas | 4.117974 |
| CK_1d | Lysinibacillus | 3.005008 |
| CK_1d | Pseudoxanthomonas | 2.058987 |
| CK_1d | Unassigned | 2.058987 |
| CK_1d | Ochrobactrum | 1.613801 |
| CK_1d | Gracilibacillus | 0.33389 |
| CK_1d | Rhodococcus | 0.166945 |
| 2%Ca_15 | Bacillus | 26.35897 |
| 2%Ca_15 | Streptomyces | 20.30769 |
| 2%Ca_15 | others | 16.82051 |
| 2%Ca_15 | Unassigned | 8.410256 |
| 2%Ca_15 | Rhodococcus | 5.435897 |
| 2%Ca_15 | Stenotrophomonas | 4.512821 |
| 2%Ca_15 | Gracilibacillus | 3.794872 |
| 2%Ca_15 | Microvirga | 2.769231 |
| 2%Ca_15 | Phenylobacterium | 2.769231 |
| 2%Ca_15 | Devosia | 2.461538 |
| 2%Ca_15 | Cellulosimicrobium | 2.358974 |
| 2%Ca_15 | Sphingomonas | 1.333333 |
| 2%Ca_15 | Pseudoxanthomonas | 1.025641 |
| 2%Ca_15 | Lysinibacillus | 0.820513 |
| 2%Ca_15 | Oceanobacillus | 0.410256 |
| 2%Ca_15 | Ochrobactrum | 0.410256 |
| 2%Ca_30 | Bacillus | 37.34756 |
| 2%Ca_30 | Streptomyces | 16.61585 |
| 2%Ca_30 | others | 13.87195 |
| 2%Ca_30 | Ochrobactrum | 10.21341 |
| 2%Ca_30 | Pseudoxanthomonas | 10.21341 |
| 2%Ca_30 | Lysinibacillus | 5.640244 |
| 2%Ca_30 | Unassigned | 1.52439 |
| 2%Ca_30 | Oceanobacillus | 1.067073 |
| 2%Ca_30 | Sphingomonas | 1.067073 |
| 2%Ca_30 | Gracilibacillus | 0.914634 |
| 2%Ca_30 | Rhodococcus | 0.609756 |
| 2%Ca_30 | Stenotrophomonas | 0.457317 |
| 2%Ca_30 | Cellulosimicrobium | 0.152439 |
| 2%Ca_30 | Devosia | 0.152439 |
| 2%Ca_30 | Microvirga | 0.152439 |
| 2%Ca_30 | Phenylobacterium | 0 |
| 3%Ca_15 | others | 25.1932 |
| 3%Ca_15 | Bacillus | 20.40185 |
| 3%Ca_15 | Unassigned | 18.7017 |
| 3%Ca_15 | Streptomyces | 12.05564 |
| 3%Ca_15 | Rhodococcus | 6.18238 |
| 3%Ca_15 | Cellulosimicrobium | 4.173107 |
| 3%Ca_15 | Gracilibacillus | 3.554869 |
| 3%Ca_15 | Devosia | 3.24575 |
| 3%Ca_15 | Stenotrophomonas | 2.009274 |
| 3%Ca_15 | Microvirga | 1.545595 |
| 3%Ca_15 | Oceanobacillus | 0.618238 |
| 3%Ca_15 | Ochrobactrum | 0.618238 |
| 3%Ca_15 | Phenylobacterium | 0.618238 |
| 3%Ca_15 | Lysinibacillus | 0.463679 |
| 3%Ca_15 | Sphingomonas | 0.463679 |
| 3%Ca_15 | Pseudoxanthomonas | 0.15456 |
| 3%Ca_30 | Bacillus | 29.06667 |
| 3%Ca_30 | Ochrobactrum | 23.73333 |
| 3%Ca_30 | Pseudoxanthomonas | 22.93333 |
| 3%Ca_30 | Streptomyces | 7.2 |
| 3%Ca_30 | others | 5.866667 |
| 3%Ca_30 | Lysinibacillus | 4.266667 |
| 3%Ca_30 | Unassigned | 2.4 |
| 3%Ca_30 | Cellulosimicrobium | 1.333333 |
| 3%Ca_30 | Gracilibacillus | 1.066667 |
| 3%Ca_30 | Stenotrophomonas | 1.066667 |
| 3%Ca_30 | Sphingomonas | 0.533333 |
| 3%Ca_30 | Devosia | 0.266667 |
| 3%Ca_30 | Oceanobacillus | 0.266667 |
| 3%Ca_30 | Microvirga | 0 |
| 3%Ca_30 | Phenylobacterium | 0 |
| 3%Ca_30 | Rhodococcus | 0 |

| MRB | | |
| --- | --- | --- |
| CK_1d | Bacillus | 19.36364 |
| CK_1d | Ensifer | 15.72727 |
| CK_1d | others | 12.90909 |
| CK_1d | Pseudomonas | 8.727273 |
| CK_1d | Streptomyces | 8.727273 |
| CK_1d | Pseudoxanthomonas | 7.272727 |
| CK_1d | Lysinibacillus | 5.727273 |
| CK_1d | Unassigned | 4.545455 |
| CK_1d | Rhodococcus | 4.272727 |
| CK_1d | Devosia | 4.090909 |
| CK_1d | Mesorhizobium | 3.909091 |
| CK_1d | Nocardia | 2.818182 |
| CK_1d | Sphingomonas | 1.181818 |
| CK_1d | Microvirga | 0.272727 |
| CK_1d | Ochrobactrum | 0.272727 |
| CK_1d | Rhizobium | 0.181818 |
| 2%Ca_15 | Unassigned | 36.65835 |
| 2%Ca_15 | others | 14.96259 |
| 2%Ca_15 | Bacillus | 6.733167 |
| 2%Ca_15 | Devosia | 6.234414 |
| 2%Ca_15 | Pseudoxanthomonas | 6.234414 |
| 2%Ca_15 | Pseudomonas | 5.486284 |
| 2%Ca_15 | Rhizobium | 5.486284 |
| 2%Ca_15 | Rhodococcus | 5.236908 |
| 2%Ca_15 | Microvirga | 4.488778 |
| 2%Ca_15 | Ensifer | 2.743142 |
| 2%Ca_15 | Nocardia | 2.743142 |
| 2%Ca_15 | Streptomyces | 1.496259 |
| 2%Ca_15 | Sphingomonas | 0.997506 |
| 2%Ca_15 | Mesorhizobium | 0.249377 |
| 2%Ca_15 | Ochrobactrum | 0.249377 |
| 2%Ca_15 | Lysinibacillus | 0 |
| 2%Ca_30 | Bacillus | 26.92308 |
| 2%Ca_30 | Unassigned | 16.51584 |
| 2%Ca_30 | others | 14.70588 |
| 2%Ca_30 | Pseudoxanthomonas | 11.53846 |
| 2%Ca_30 | Lysinibacillus | 10.181 |
| 2%Ca_30 | Ochrobactrum | 9.049774 |
| 2%Ca_30 | Ensifer | 2.262443 |
| 2%Ca_30 | Mesorhizobium | 2.262443 |
| 2%Ca_30 | Microvirga | 1.58371 |
| 2%Ca_30 | Rhizobium | 1.58371 |
| 2%Ca_30 | Sphingomonas | 1.357466 |
| 2%Ca_30 | Pseudomonas | 0.904977 |
| 2%Ca_30 | Streptomyces | 0.678733 |
| 2%Ca_30 | Devosia | 0.452489 |
| 2%Ca_30 | Nocardia | 0 |
| 2%Ca_30 | Rhodococcus | 0 |
| 3%Ca_15 | Unassigned | 24.07407 |
| 3%Ca_15 | others | 19.62963 |
| 3%Ca_15 | Devosia | 8.518519 |
| 3%Ca_15 | Bacillus | 7.777778 |
| 3%Ca_15 | Pseudomonas | 7.407407 |
| 3%Ca_15 | Pseudoxanthomonas | 6.296296 |
| 3%Ca_15 | Rhizobium | 5.925926 |
| 3%Ca_15 | Rhodococcus | 5.925926 |
| 3%Ca_15 | Microvirga | 4.814815 |
| 3%Ca_15 | Sphingomonas | 4.444444 |
| 3%Ca_15 | Ensifer | 2.222222 |
| 3%Ca_15 | Nocardia | 2.222222 |
| 3%Ca_15 | Streptomyces | 0.740741 |
| 3%Ca_15 | Lysinibacillus | 0 |
| 3%Ca_15 | Mesorhizobium | 0 |
| 3%Ca_15 | Ochrobactrum | 0 |
| 3%Ca_30 | Bacillus | 37.2 |
| 3%Ca_30 | Pseudoxanthomonas | 19 |
| 3%Ca_30 | others | 12.8 |
| 3%Ca_30 | Ochrobactrum | 10.8 |
| 3%Ca_30 | Lysinibacillus | 7.4 |
| 3%Ca_30 | Unassigned | 7.4 |
| 3%Ca_30 | Mesorhizobium | 1.6 |
| 3%Ca_30 | Devosia | 0.8 |
| 3%Ca_30 | Ensifer | 0.8 |
| 3%Ca_30 | Pseudomonas | 0.8 |
| 3%Ca_30 | Microvirga | 0.6 |
| 3%Ca_30 | Sphingomonas | 0.6 |
| 3%Ca_30 | Streptomyces | 0.2 |
| 3%Ca_30 | Nocardia | 0 |
| 3%Ca_30 | Rhizobium | 0 |
| 3%Ca_30 | Rhodococcus | 0 |

**Table. S3 Feasibility Comparative Analysis of Four Carbon Sources**

| Carbon Source | Cost | Availability | Toxicity |
| --- | --- | --- | --- |
| p-Hydroxy-phenylacetic acid | High cost | Moderate difficulty | Low toxicity |
| β-Methyl-D-glucoside | Moderate-high | Moderate difficulty | Low toxicity |
| D-Mannitol | Low cost | Easy to obtain | Low toxicity |
| D-Aspartic acid | Moderate cost | Easy to obtain | Low toxicity |

**Fig. S1: Effect of calcium peroxide on soil organic matter.** **CaO₂ was applied at three concentrations (1%, 2%, and 3%). soil1, soil2, and soil3 represent soil samples with three different levels of soil organic matter.**

**Fig. S2:** **The comprehensive effects of CaO_2_ treatment on the four types of soil ARB were analyzed. Without distinguishing between treatment gradients and ARB types, the overall changes in soil ARB were examined across three time points: day 0, day 15, and day 30.**

**Fig. S3: Layout of assays in the MicroPlate**

**Fig. S4: Removal of four types of ARB by CaO_2_ treatment for 10 days followed by adding 1 mM D-Mannitol. The four types of soil ARB are SRB (sulfonamide-resistant bacteria), TRB (tetracycline-resistant bacteria), MRB (macrolide-resistant bacteria), and BRB (beta-lactam-resistant bacteria).**
